# Effects of Magnetostatic Interactions in FeNi-Based Multilayered Magnetoimpedance Elements

**DOI:** 10.3390/s24196308

**Published:** 2024-09-29

**Authors:** Grigory Yu. Melnikov, Sergey V. Komogortsev, Andrey V. Svalov, Alexander A. Gorchakovskiy, Irina G. Vazhenina, Galina V. Kurlyandskaya

**Affiliations:** 1 Institute of Natural Sciences and Mathematics, Ural Federal University, 620002 Ekaterinburg, Russia; grigory.melnikov@urfu.ru (G.Y.M.); andrey.svalov@urfu.ru (A.V.S.); 2 Kirensky Institute of Physics, Federal Research Center SB RAS, 660036 Krasnoyarsk, Russia; komogor@iph.krasn.ru (S.V.K.); vigetch@list.ru (A.A.G.) irina-vazhenina@mail.ru (I.G.V.); 3 Polytechnic School, Siberian Federal University, 660041 Krasnoyarsk, Russia

**Keywords:** magnetic multilayers, permalloy, magnetic properties, magnetic domains, magnetostatic interactions, ferromagnetic resonance, magnetoimpedance, magnetic field sensors

## Abstract

Multilayered [Cu(3 nm)/FeNi(100 nm)]_5_/Cu(150 nm)/FeNi(10 nm)/Cu(150 nm)/FeNi(10 nm)/Cu(150 nm)/[Cu(3 nm)/FeNi(100 nm)]_5_ structures were obtained by using the magnetron sputtering technique in the external in-plane magnetic field. From these, multilayer magnetoimpedance elements were fabricated in the shape of elongated stripes using the lift-off lithographic process. In order to obtain maximum magnetoimpedance (MI) sensitivity with respect to the external magnetic field, the short side of the rectangular element was oriented along the direction of the technological magnetic field applied during the multilayered structure deposition. MI sensitivity was defined as the change of the total impedance or its real part per unit of the magnetic field. The design of the elements (multilayered structure, shape of the element, etc.) contributed to the dynamic and static magnetic properties. The magnetostatic properties of the MI elements, including analysis of the magnetic domain structure, indicated the crucial importance of magnetostatic interactions between FeNi magnetic layers in the analyzed [Cu(3 nm)/FeNi(100 nm)]_5_ multilayers. In addition, the uniformity of the magnetic parameters was defined by the advanced technique of the local measurements of the ferromagnetic resonance field. Dynamic methods allowed investigation of the elements at different thicknesses by varying the frequency of the electromagnetic excitation. The maximum sensitivity of 40%/Oe with respect to the applied field in the range of the fields of 3 Oe to 5 Oe is promising for different applications.

## 1. Introduction

Magnetic soft multilayered structures are relevant in numerous technological applications. Modern electronics are developing in the direction of patterned multilayered films with different shaped electronic components [1,2,3,4]. One of the perspective applications is the fabrication of sensitive elements for detecting small magnetic fields up to the signals corresponding to the stray fields of magnetic labels or biomagnetic signals from living systems [5,6,7,8]. Magnetoimpedance (MI) is one of the most promising effects on which sensitive magnetic detectors can be based. Its advantage is its highest sensitivity with respect to external applied magnetic fields observed in a low field in the order of a few Oersted at room temperature [9,10,11,12].

The MI effect is the change of the ferromagnetic conductor impedance (Z) when high-frequency current flows through it and an external magnetic field is applied. This is a classical electrodynamic effect related to the dependence of the skin penetration depth (δ) on the dynamic magnetic permeability (µ) [13,14]. The skin penetration depth can be estimated as δ = (πfσμ)^−0.5^, where f is the driving current frequency, and σ is the conductivity of the material. As can be seen, frequency is a significant parameter; the lower frequency is more acceptable for technical applications. On the other hand, low frequency leads to a higher skin penetration depth; consequently, the thickness of the material should be higher. A high sensitivity of MI elements is observed for a frequency of the order of tens of MHz at a thickness of about half of the micron of the magnetic layers.

Permalloy (Fe_20_Ni_80_) is a magnetically soft material used in most sensor applications [14,15,16,17]. However, at a thickness of about 100 nm transition, an in “transcritical” state is observed. This state of permalloy is characterized by an increase in the coercivity H_c_ and particular features of the shape of the magnetic hysteresis loop, stripe magnetic domains formation, and appearance of “rotational anisotropy” [18,19]. To avoid this effect, the concept of nanostructuring was proposed [18,19,20]. Nanostructuring is the separation of magnetic layers of permalloy by a conductive material [21,22]. Copper is frequently a useful material due to availability, high conductivity, and ease of etching in the course of the standard techniques of electronic component production. The thickness of the cooper spacers in MI multilayered structures has been analyzed previously and, for the present studies, we just took into account existing references [19,20,21,22,23]. However, the analysis of magnetostatic interactions between magnetic FeNi layers in [Cu(3 nm)/FeNi(100 nm)]_5_ multilayered structures is absent in the literature.

Based on a previous research, reports of high magnetoimpedance effects are observed in symmetric structures, with two layers of [Cu(3 nm)/FeNi(100 nm)]_5_ separated by 500 nm Cu [22,23,24]. The MI element requires a stripe shape for measurements in “microstripe” lines and induced magnetic anisotropy perpendicular to the short side of the stripe. In this case, the highest magnetoimpedance is observed when a high frequency current and magnetic field are directed along the long side of the element [24,25]. Although magnetoimpedance is high for this type of structure, it is still lower in comparison with theoretical predictions. One reason for the low MI effect is the inhomogeneity of the magnetic properties in the FeNi layers, i.e., the magnetic anisotropy field, the dispersion of induced magnetic anisotropy axes, and magnetization. Also, other complicated contributions (for instance, magnetostatic interactions between magnetic layers) can be essential. The asymmetry of the magnetic properties of the magnetic layers is due to a long deposition time and the presence of a thick Cu layer in the middle. Previously [26], we have shown that a 0.5 μm thick Cu lead has a rather high average grain size up to 50 nm in comparison with the typical size of 12–25 nm for a thin FeNi film of 100 nm. The grain size and texture of the Cu lead usually contribute to the structural peculiarities of the FeNi layer immediately above the Cu lead and, consequently, to the properties of the other layers of the top multilayered structure. As a result, the structure and the magnetic properties of the top and bottom multilayers differ from each other and the asymmetry of their properties become reflected in the decrease in MI. In a previous work [27], we suggested that the problem of magnetic property asymmetries related to thick Cu leads [26] can be partially solved using the nanostructuring of FeNi layers by Cu sublayers. However, this complex technological step requires further investigation.

Understanding magnetic properties with inhomogeneities like multilayered structures is an actual task. Static methods such as vibrating sample magnetometry (VSM) and Kerr microscopy give signals from a whole sample or a thin surface area (about 20 nm for a Kerr microscope). Indirect dynamic magnetic methods such as ferromagnetic resonance (FMR) and MI effect related to dynamic magnetic permeability allow set investigation thickness (skin penetration depth) by variation of frequency. Resonance spectra for FMR are analyzed by widely used Sul–Smith equations [28,29], while, for MI effects, a more complicated system using the Landau–Lifshitz equation with special boundary conditions is calculated [30,31,32]. These theoretical approximations estimate magnetic anisotropy fields, dispersion of induced magnetic anisotropy axes, magnetization, etc. 

The measurements of the impedance Z and its active (R) and reactive (X) components (Z(f) = R(f) − iX(f)) were widely discussed in the literature [33,34]. However, for practical applications in certain conditions devices with active part R control working principle may have an advantage [35].

In this work, FeNi/Cu-based multilayered MI rectangular elements separated by Cu lead were obtained by a standard lift-off lithographic process. Elements were investigated by static (VSM, Kerr microscopy) and dynamic (FMR, MI effect) methods in order to estimate magnetic anisotropy field, dispersion of induced magnetic anisotropy axes, and magnetization at different thicknesses, revealing the crucial role of the magnetostatic interactions between FeNi layers in [Cu(3 nm)/FeNi(100 nm)]_5_ multilayered structures.

## 2. Experiment

**Figure 1:**
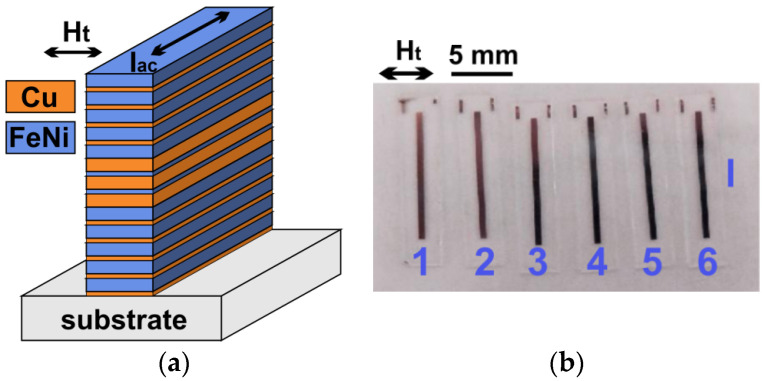
Scheme of multilayered [Cu(3 nm)/FeNi(100 nm)]_5_/Cu (150 nm)/[FeNi (3 nm)/Cu(150 nm)/[FeNi (3 nm)/Cu(150 nm)/[FeNi(100 nm)/Cu(3 nm) ]_5_ element in magnetoimpedance geometry. Ht—direction of the application of technological magnetic field during multilayered structure deposition. Iac—direction of the flow of the high frequency not alternating current during magnetoimpedance applications. Note that the structures are shown in their real scale (**a**). Photograph of 1–6 lithographic MI element arrangements; l is a length of 10 mm (**b**).

Multilayered [Cu(3 nm)/FeNi(100 nm)]_5_/Cu(150 nm)/FeNi(10 nm)/Cu(150 nm)/FeNi(10 nm)/Cu(150 nm)/[Cu(3 nm)/FeNi(100 nm)]_5_ MI elements were obtained by dc magnetron sputtering onto Corning glass substrates at room temperature. Parameters of deposition were next, with a background pressure of 3 × 10^−7^ mbar and a working argon pressure of 3.8 × 10^−3^ mbar. The thickness of the layers was defined by the deposition time using previously calibrated rates. For fabrication, a batch of the magnetoimpedance sensitive elements of a standard optical lift-off lithography was employed [36]. The obtained magnetoimpedance elements were configured with an open magnetic flux [26], i.e., the rectangular elements consisted of a number of layers of the same width and length (0.5 mm × 10.0 mm) organized as a vertical structure (Figure 1a).

Two batches of twelve elements (batch I and batch II) were arbitrarily selected for characterization by different techniques. Therefore, the denomination of the elements included the batch number (I or II), and position (from 1 to 6) (Figure 1b). MI element thicknesses for magnetic properties estimation were measured by sharp step and analyzed with a Dektak 150 stylus profilometer (Veeco, Somerset, NJ, USA). The thickness of all samples was (1.20 ± 0.04) µm. During the deposition process, an in-plane constant technological magnetic field Ht = 100 Oe was applied along the short side of the MI elements in order to induce a transverse uniaxial in-plane magnetic anisotropy.

Static magnetic properties measurements were carried out by the means of a 7407 VSM vibrating sample magnetometer (Lake Shore Cryotronics, London, UK) and a magneto-optical Kerr effect (MOKE) using a specialized optical microscope Evico (Evico, Dresden, Germany). The latter equipment was also used for the magnetic domain structure observation in different external magnetic fields applied in the plane of the MI element. 

A rectangular multilayered MI sensitive element was placed into a “microstripe” line, which was contacted by highly conductive silver painting on the short sides. A uniform constant external magnetic field (H) up to 100 Oe was created by a pair of Helmholtz coils. It was applied along the long side of the rectangular element and therefore the longitudinal magnetoimpedance configuration was employed. The alternating current flowed parallel to the external magnetic field, providing the highest sensitivity of the MI ratio. 

The total impedance was measured by a network analyzer (Agilent E8358A) in a frequency range of 0.1–400 MHz, with 1 mA amplitude of the excitation current across the multilayered element. The calibration and mathematical subtraction of the test fixture contribution procedures were performed in accordance with previously well-described procedures. The MI ratio and MI ratio sensitivity for the resistive component of impedance were calculated as follows:

(1) $$\Delta R/R\left(H\right)=\frac{R\left(H\right)-R(H_{max})}{R(H_{max})}\cdot 100\%$$

where R(H) and R(H_max_) are the resistances corresponding to the external magnetic fields H and H_max_, respectively. The magnetic field sensitivity of the MI ratio, i.e., the change of the real component of the impedance ratio per unit of the external magnetic field, was determined by the following expression:

(2) $$s\left(\Delta R/R\right)=\frac{\Delta R/R}{\Delta H}$$

where ΔH = 0.1 Oe is the increment for an external magnetic field.

**Figure 2:**
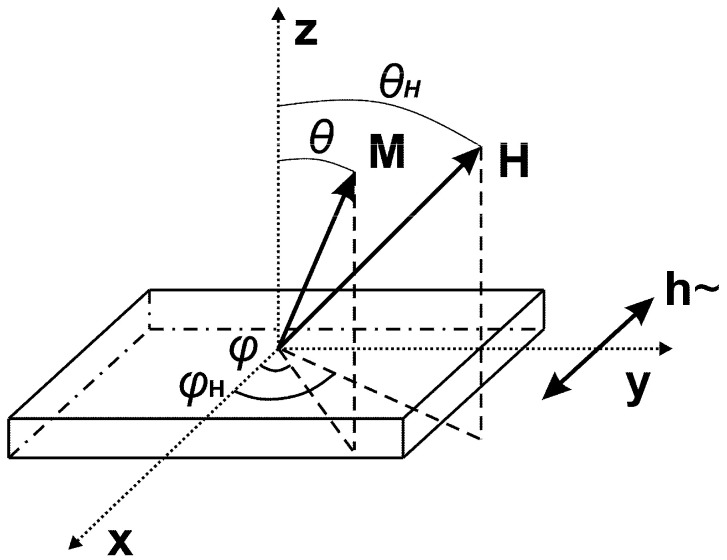
Scheme of FMR measurements. Here, M is the magnetization vector, H is an external constant magnetic field, and h is a microwave rf field. For definition of all angles, see also the main text.

The ferromagnetic resonance (FMR) of the MI multilayered structures was studied on the basis of the measurements of absorption spectra by a home-made scanning FMR spectrometer (Kirensky Institute of Physics, Krasnoyarsk, Russia) using a microstrip resonator with a hole 0.8 mm in diameter [37]. This means that high frequency properties were measured from the local area of about 1 mm in diameter. FMR spectra were measured at a fixed frequency 1.3 GHz and at room temperature. The measurements were carried out with the direction of the external constant magnetic field Hc hanging in the film plane, i.e., in in-plane configuration (the angle between the magnetization M and the applied constant field was variable) (Figure 2).

In all configurations, the radio frequency alternating magnetic field (ac) magnetic field h was perpendicular to the external constant magnetic field, i.e., h~⊥ H. The longitudinal MI effect employed in this work (according to the literature it is the most useful configuration for applications) also dealt with the same configuration of the external magnetic field and the direction of the flow of the alternating current. FMR is an indirect method for determining the magnetic characteristic of magnetic thin film. It is based on the investigation of resonance field depending on the angle at the in-plane (*θ_H_* = 90°) or out-of-plane (*φ_H_* = const) configuration.

The resonance field at fixed *θ_H_* and *φ_H_* angles for homogeneous film could be defined by an equation of motion of magnetization:

(3) $${\left(\frac{\omega }{\gamma }\right)}^{2}=\frac{1}{{M_{eff}}^{2}\cdot {sin}^{2}(\theta )}\cdot \left(\frac{\partial^{2}E}{\partial \theta^{2}}\cdot \frac{\partial^{2}E}{\partial \varphi^{2}}-{\left(\frac{\partial^{2}E}{\partial \varphi \partial \theta }\right)}^{2}\right)$$

Taking into account the magnetization equilibrium state [27,28,29].

(4) $$\frac{\partial E}{\partial \varphi }=\frac{\partial E}{\partial \theta }=0$$

where γ = 1.758 × 10^7^ Hz/Oe is the gyromagnetic ratio; *ω* is the resonance frequency; *E* is the free energy density. The equation of free energy density includes the following: *E_H_* is the energy of Zeeman; *E_nz_* is the demagnetization field perpendicular to the plane; *E_u_* is the induced uniaxial magnetic anisotropy in-plane (*K_u_* = *H_a_* × *M_eff_*/2); *E_nxy_* is the energy of demagnetization in-plane of the film [38,39,40].

(5) $$E=E_{H}+E_{nz}+E_{u}+E_{nxy}\;E_{H}=-M_{eff}\cdot H\cdot (\sin\left(\theta \right)\cdot \sin\left(\theta_{H}\right)\cdot \cos\left(\varphi -\varphi_{H}\right)+\cos\left(\theta \right)\cdot \cos\left(\theta_{H}\right))\;E_{nz}=\frac{N_{z}}{2}{\cdot M_{eff}}^{2}{\cdot cos}^{2}(\theta )\;E_{u}=-K_{u}\cdot {sin}^{2}(\theta )\cdot {cos}^{2}(\varphi -\varphi_{0})\;E_{nxy}=\frac{{M_{eff}}^{2}}{2}(N_{x}\cdot {cos}^{2}\left(\varphi \right)+N_{y}\cdot {sin}^{2}\left(\varphi \right))\cdot {sin}^{2}(\theta )$$

where *θ* is theangle between the *z*-axis and magnetization; *θ_H_* is the angle between the *z*-axis and the external magnetic field; *H* is the external magnetic field; *M_eff_* is the effective magnetization; *φ* is the angle between the *x*-axis and magnetization; *φ_H_* is the angle between the *x*-axis and the external magnetic field; *φ*_0_ is the angle between the *x*-axis and the magnetic anisotropy axes; *N_x_*, *N_y_*, and *N_z_* are the demagnetization factors; *K_u_* is the constant of induced uniaxial magnetic anisotropy (*K_u_* = *H_a_* × *M_eff_*/2).

In this work, the in-plane configuration of FMR (*θ_H_* = 90°) was measured. Therefore, the equilibrium direction of magnetization lay in-plane (*θ* = 90°) due to the influence of the demagnetization field being perpendicular to the plane. Varied parameters were effective magnetization *M_eff_* and magnetic anisotropy field *H_a_*. The parameters *M_eff_* and *H_a_* were varied until the experimental curve matched the approximation curve with an accuracy of 5% or less. Local measurements of ferromagnetic resonance field were made using a homemade scanning FMR spectrometer and a microstrip resonator with a hole 0.8 mm in diameter. This device allowed to measure the heterogeneities of the magnetic properties along the long side of MI elements.

The measurements of MI effects and FMR spectra were carried out at a high frequency, so it was important to take into account skin effect. The advantage of skin effect for magnetodynamic methods is the opportunity to measure magnetic properties of a local area of the sample with a thickness equal to the skin depth. Skin penetration depth can be estimated by Equation [41]:

(6) $$\delta =c\;\cdot \sqrt{\frac{1}{4\pi^{2}\cdot f\cdot \mu (f)\cdot \sigma \;}}$$

where *σ* is the conductivity, *f* is the current frequency, *µ*(*f*) is the transverse magnetic permeability at resonance frequency, and *c* is the speed of light. 

Let us estimate the skin penetration depth for frequencies at which measurements were carried out. For this, evaluate transverse magnetic permeability for MI elements investigated in this paper (6): effective magnetization *M_eff_* = 750 G, thickness *d* = 1 µm, permalloy conductivity *σ* = 3 × 10^16^ s^−1^, Gilbert damping parameter *κ* = 0.02, magnetic anisotropy field *Ha* = 7 Oe, *ψ* -deviation of the magnetic anisotropy axis from the transverse direction [31].

(7) $$\mu =1+\frac{\gamma 4\pi M_{eff}\left(\gamma 4\pi M_{eff}+\omega_{1}-i\kappa \omega \right){sin}^{2}\theta }{\left(\gamma 4\pi M_{eff}+\omega_{1}-i\kappa \omega \right)\left(\omega_{2}-i\kappa \omega \right)-\omega^{2}},$$

$$\omega_{1}=\gamma [H_{a}{cos}^{2}\left(\theta -\psi \right)+Hsin\theta ]$$

$$\omega_{2}=\gamma [H_{a}cos\left(2(\theta -\psi )\right)+Hsin\theta ]$$

$$H_{a}\sin\left(\theta -\psi \right)\cos\left(\theta -\psi \right)=Hcos\theta$$

In the case of the MI effect, when the frequency of the current changes from 100 MHz to 400 MHz, the minimum skin depth varies from 600 nm to 300 nm and is observed in the field of magnetic anisotropy *H_a_* = 7 Oe. With a further increase in frequency to 1.3 GHz in the case of FMR, the skin penetration depth in the resonance field from 15 Oe to 30 Oe is about 200 nm. Since, at the measurements of the MI effect and FMR, the penetration of the exciting electromagnetic field occurs from two surfaces of multilayered element structures, the signal actually comes from a thickness equal to twice the skin penetration depth. 

## 3. Results and Discussion

### 3.1. Static Magnetic Properties

Static magnetic properties were investigated by a VSM magnetometer and a magneto-optical Kerr microscope. The VSM magnetometer allowed to investigate the magnetic properties of the whole sample, including quantitative estimates of magnetization. The Kerr microscopy was associated with the properties of the surface layer in the case of FeNi and visualized the magnetic domain structure.

**Figure 3:**
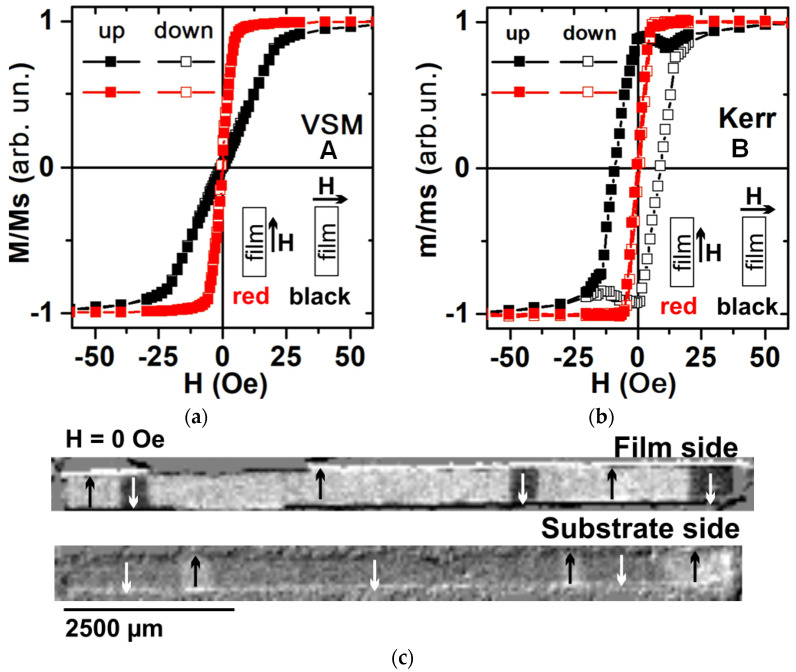
VSM (**a**) and magneto-optical Kerr (**b**) in-plane magnetic hysteresis loops measured along the long (red curves) and short (black curves) sides of MI elements. Magnetic domain images obtained from both sides of the MI element A at zero magnetic field (**c**). Here “up” is for the measurements increasing in the external field starting from the saturation in the maximum negative field and “down” is for the measurements decreasing in the external field starting from the saturation in the maximum positive external field.

According to the VSM magnetic hysteresis loops, the MI elements had the coercive force value of about *Hc* = 1 Oe, and the anisotropy field was estimated as a field close to saturation *Ha* = 5 Oe. Although, during the deposition the technical magnetic field was applied along the short side of the elements, the easy magnetization axis (EMA) was along long side of the element. This behavior related to the shape anisotropy, which created an additional demagnetizing field directed along the short side of the MI elements. However, the axis of uniaxial magnetic anisotropy along the short side was confirmed by the Kerr microscope, which showed that the magnetization process occurred by magnetic domain wall displacement in this direction (Figure 3). To estimate the shape of the MI elements for calculating the demagnetizing field, an infinitely long prism was confirmed, with a thickness of t = 500 nm and width of *w* = 500 µm, for which the demagnetizing factors were *N_x_* = 0.027; *N_y_* ≈ 0; *N_z_* ≈ 12.539 (*N_x_*, along the short side; *N_y_*, along the long side; *N_z_*, perpendicular to the plane) [38,39]. 

This choice was due to the following factors. The length of the elements exceeded the width by several orders of magnitude, so the demagnetizing factor along the long side could be neglected, which corresponded to a prism with infinite length. In a multilayer structure separated by interlayers of Cu, the demagnetizing fields would be less than in a non-separated structure, i.e., the greater the thickness of the interlayers, the smaller these fields. In this case, it was assumed that the magnetostatic interaction between two multilayers separated by a thick Cu layer was negligible, and the magnetostatic interaction between the FeNi layers in each multilayer was the same as if they were not separated by Cu interlayers. Then, to estimate the demagnetizing fields, we could take the demagnetization factor for a thin film with a thickness of t = 500 nm equal to the thickness of one FeNi multilayer. It is worth noting that, for bodies of a non-ellipsoidal shape, the demagnetization factor depended in a complex way not only on the shape but also on the magnetic properties of the material, the magnetic state of the body, the distribution of magnetization in the sample, and the coordinates of the observation point. Therefore, for the estimation, we considered the state of magnetic saturation, and, by demagnetizing factor, we meant its average value over the volume (magnetometric demagnetizing factor) [38,39]. So, the estimation for the demagnetization field resulted in the value of about Hd = 22 Oe for the saturation state of the permalloy, with Ms = 820 Gs. 

The magnetization process along the long side of the MI elements according to the Kerr microscopy occurred by magnetization rotation for the anisotropy field of about Ha = 7 Oe. The VSM and Kerr microscopy magnetic hysteresis loops for magnetization along the long side were similar. Thus, the magnetization processes mainly occurring by magnetization rotation were very close for the surface layer and the whole sample (Figure 3a,b, “red”). However, the magnetization process along the short side of the MI element was different for the whole sample (Figure 3a, “black”) and the surface layer (Figure 3b, “black”), which pointed out the strong effects of magnetostatic interaction between FeNi layers and shape anisotropy.

First of all, at H = 0 Oe, according to the VSM measurements, the whole element had zero magnetization. However, according to the Kerr microscopy data, the individual magnetic FeNi layer was close to magnetic saturation along the short side (Figure 3a,b, “black”). This was possibly due to the “antiparallel” ordering of magnetic moments in the adjacent FeNi layers leading to a closed magnetic flux state.

**Figure 4:**
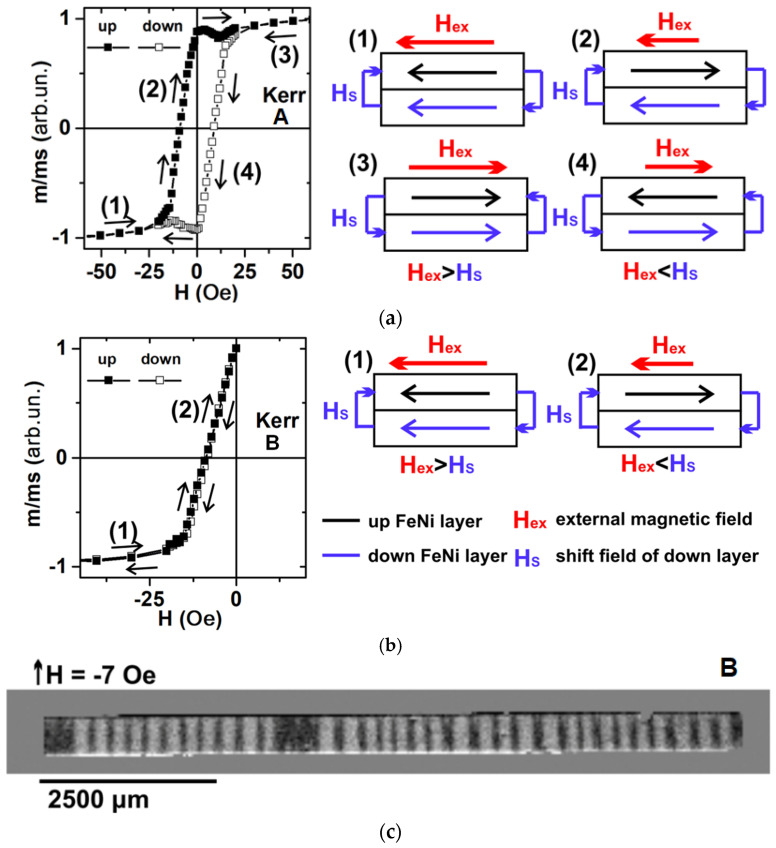
Kerr microscopy surface magnetic hysteresis loops measured along the short side of MI elements with the scheme of magnetization of elements: (**a**) magnetization from −100 Oe to 100 Oe and back; (**b**) magnetization from −100 Oe to 0 Oe and back; (**c**) image of magnetic domains of MI element corresponding to the external magnetic field H_ext_ = 7 Oe. Here “up” is for the measurements increasing in the external field starting from the saturation in the maximum negative field and “down” is for the measurements decreasing in the external field starting from the saturation in the maximum positive external field. Orientation of the external magnetic field is indicated by the red arrow; orientation of the magnetization of the top layer is indicated by the black arrow; orientation of the magnetization of the bottom layer is indicated by the blue arrow.

It was confirmed by the magnetic domain images of the free FeNi layer and the FeNi layer closest to the glass substrate at zero magnetic field that they were almost magnetically saturated having an opposite direction of magnetization (Figure 3c). The above observed behavior indicated not only an induced magnetic anisotropy but also a magnetostatic interaction between layers played an important role in the formation of effective transverse magnetic anisotropy with the axis oriented along the short side, which was necessary for the high sensitivity of the magnetoimpedance effect. Secondly, the magnetic hysteresis loop corresponding to the surface layer measured by Kerr microscopy had a “rectangular” shape. However, it described a rather complex and “non-classical” magnetic hysteresis process. Re-magnetization began when the external magnetic field was still directed along the current magnetization vector of the layer (Figure 4a). 

This feature was due to magnetic shift field *Hs*, which the FeNi bottom layer created as a result of the magnetostatic interaction, which became very important in the analyzed [Cu(3 nm)/FeNi(100 nm)]_5_ multilayered structure. The whole magnetization process can be described as follows (Figure 4a). At point (1), the magnetizations of the top and bottom FeNi layers were directed along the direction of the external magnetic field Hex. At point (2), when Hex became lower than Hs, the upper layer began with the re-magnetization process. At point (3), the value of the external field Hex became sufficient in order to change the orientation of the FeNi bottom layer, and the magnetization of the upper and bottom layers became directed along the direction of the external field. For the opposite direction of the external magnetic field, the above-described process was repeated, (point (4)). In the case under consideration, the magnetic field Hex = 7 Oe at which the magnetic moment equaled zero did not define the coercive force as for classic hysteresis; this value was close to the value of the shift field Hs. For the measurement of the coercive force using the magneto-optical Kerr microscopy, it was necessary to measure the magnetic hysteresis loop without the re-magnetization of the FeNi bottom layer. Figure 4b shows such a process. In this case, the coercivity Hc = 1 Oe had the same value as for the VSM measurements.

### 3.2. Dynamic Magnetic Properties

**Figure 5:**
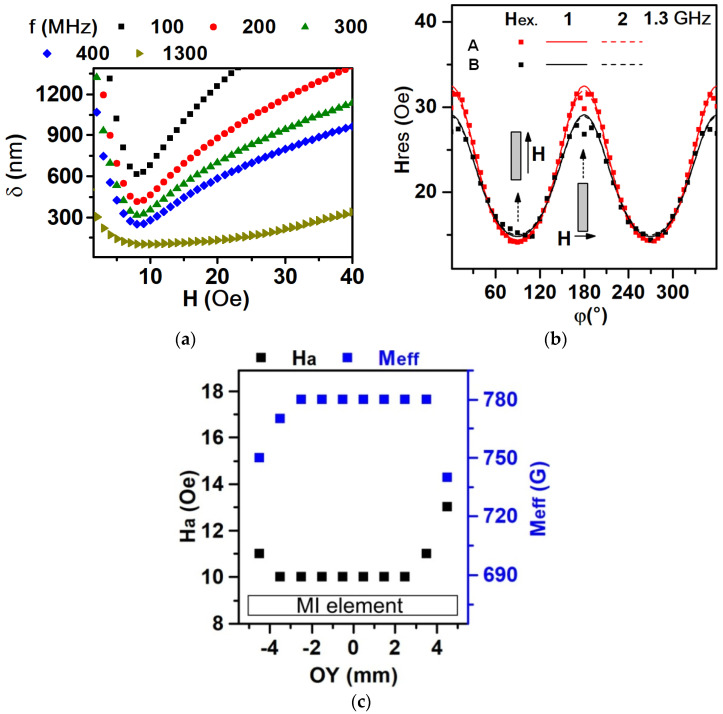
(**a**) Dependence of the skin penetration depth on the value of the magnetic field at different frequencies of the exciting electromagnetic field for the f values in the range of the experimental studies. (**b**) The angular dependence of the resonant field for MI elements A and B at a frequency of 1.3 GHz. Lines 1 and 2 are theoretical calculations of *N_x_* = 0.027 and *N_x_* = 0.022, respectively, points of the experiment. (**c**) Distribution of *H_a_* and *M_eff_* values along the long side of MI element A.

Figure 5 shows the experimental (points) and theoretical (line) dependences of the resonance fields for the MI elements denominated by A and B. The theoretical approximation was carried out with the point that the films had magnetic anisotropy along the short side of the elements according to the Kerr microscopy (*φ*_0_ = 0°) (see also Figure 3 and Figure 4). Demagnetization factors were chosen as follows: *N_x_* ≈ 0.027; *N_y_* ≈ 0; *N_z_* ≈ 12.539 (*N_x_*, along the short side; *N_y_*, along the long side; *N_z_*, perpendicular to the plane). In this case, approximation could be reached for the next varied parameters (*M_eff_* and *H_a_*), i.e., for element A *M_eff_* = 740 Gs, *H_a_* = 10 Oe; for element B *M_eff_* = 780 Gs, Ha = 14 Oe. This also confirmed the presence of induced magnetic anisotropy along the short side. The minimum of the resonance field was observed at the direction of the field being parallel to the long side of the element. This pointed out the dominant effect of the demagnetization field over the induced magnetic anisotropy at the magnetic saturation state. The Effective magnetization value was in an agreement with the values of magnetization carried out by VSM and Kerr microscopy, but the magnetic anisotropy was higher.

If we selected the demagnetizing factor *N_x_* = 0.022, a good approximation could be reached for the next varied parameters (Meff and *H_a_*), i.e., for element A *M_eff_* = 740 Gs, *H_a_* = 7 Oe; for element B *M_eff_* = 780 Gs, *H_a_*= 10 Oe. So, the value of the magnetic anisotropy field corresponded to the results of the Kerr microscopy and VSM magnetometry, while the effective magnetization remained the same. Thus, FMR can be used to estimate the demagnetization factor from other known quantities. The demagnetizing factor describing the demagnetization field could be lower due to the presence of Cu layers, which decreased the magnetostatic interaction between the FeNi layers with increasing thicknesses. 

An interesting feature was observed at an angle when the external field was applied along the short side of the element (*φ* = 180° ± 5°). There was a local minimum (Figure 5b). This complicated behavior could be explained by the following suggestions. The first suggestion is that, at a frequency of 1.3 GHz, the resonant field of about 32 Oe was not sufficient for magnetization saturation due to the influence of the demagnetization field (Figure 3a, “black”). Thus, the inhomogeneity of the resonance field was present along the element. The second suggestion is that the magnetostatic interaction between the permalloy layers (see the explanation in Section 3.1) made an additional contribution.

As the scanning FMR spectrometer consisted of a microstrip resonator with a hole of 0.8 mm in diameter, it allowed to measure the heterogeneities of the magnetic properties along the long side of the MI elements, with ane accuracy about the size of the hole. It can be seen in Figure 5c that, at the edges of the elements, the values of *M_eff_* decreased and *H_a_* increased. It could be supposed that this behavior was connected with the complicated distribution of the demagnetization fields at the edges. 

**Figure 6:**
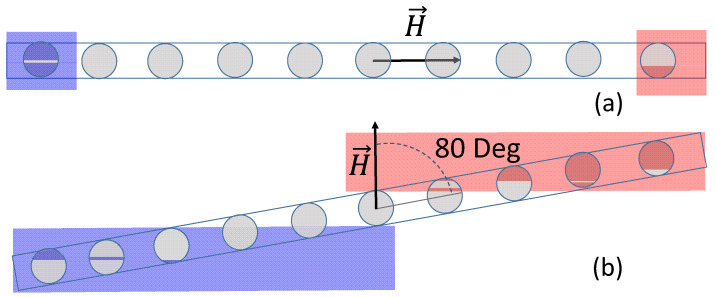
Schematic representation of the geometry of local FMR measurements for MI strip element: external magnetic field is applied along the long (**a**) and short (**b**) sides of the element. See also the main text.

The magnetoimpedance effect is another magnetodynamic effect connected with FMR. The difference between them is that energy absorption does not have resonant behavior and it is measured in the external field range near the field of saturation [42,43,44]. Energy absorption is related to the resistive component of impedance. Therefore, as the next step, we considered the real part of the MI study. The magnetoimpedance effect was measured in the “microstripe” line, in which the high frequency current flowed through the element. The current created an rf-magnetic field (*h*) like in the FMR measurements, and the resistance (energy absorption) was measured with the changing external static magnetic field (*H*). The parameters *M_eff_* and *H_a_* shown in Figure 5c were determined from the angular dependence of the FMR field taken from a certain region of the MI strip element. The position for each measurement is shown using the gray circles (Figure 6). 

This angular dependence was calculated using the Stoner–Wohlfarth model, an essential assumption of which is the homogeneity of magnetization, and the uniform rotation of magnetization within the sample. For the sample as a whole, the resonance fields (15 ÷ 32 Oe) corresponded to the saturated state of the MI strip element (see Figure 3a). In the case of applying the external magnetic field along the long axis of the MI element, the orientational homogeneity of magnetization was significantly disrupted near the edges of the strip (Figure 6a, where the regions of inhomogeneous magnetization are colored blue and pink). The deviations of *M_eff_* and *H_a_* values near the strip edges (Figure 5c) were exactly related to this. It was evident that the region of non-uniform magnetization orientation extended approximately 1 mm from each edge of the stripe. For the point in the center of the strip (from which the angular dependence shown in Figure 5b was taken), the magnetization could be considered uniform in the angular range ϕ > 10÷90 deg, but would be non-uniform at ϕ = 0 ÷ 10 deg (in Figure 6b, it corresponded with the angle between the field and the long axis of the MI element, i.e., 90 deg – ϕ = 80 deg). In this angular range, the angular dependence of the resonant field (Figure 6b) exhibited a deviation from the line corresponding to the Stoner–Wohlfarth model. In the region between the strip edges (1 mm long), Figure 5c exhibits remarkable homogeneity of the absolute values of *M_eff_* and *H_a_* along the strip length. This means that, when detecting an external field by an element of a given geometry using MI, the direction of the external field along the long axis of the element is preferable, and the length of the element significantly exceeds the length of the edge effect by ~1 mm.

**Figure 7:**
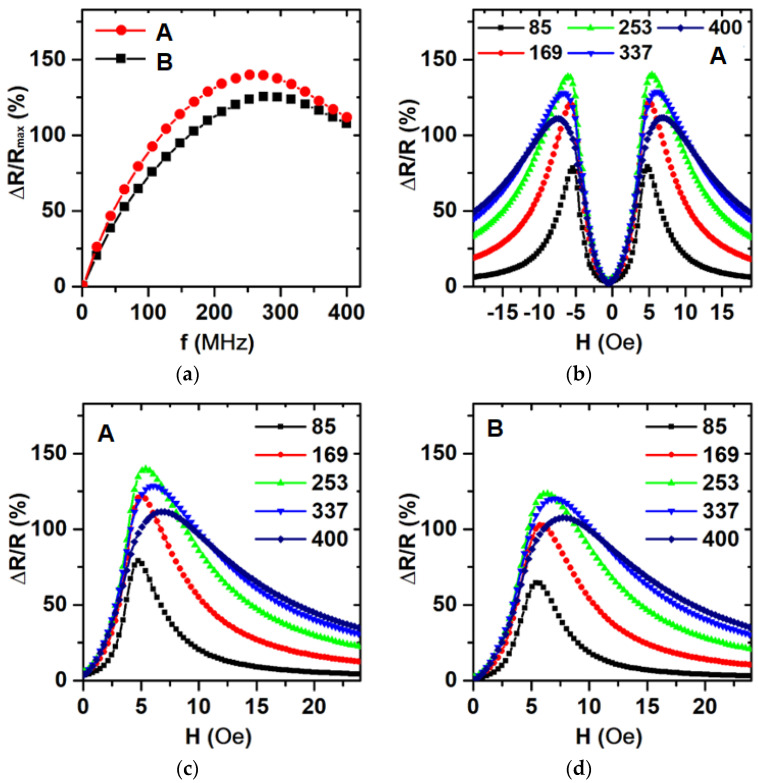
Frequency dependence of maximum MI ratio for real part of the impedance ratio (**a**); field dependence of MI ratio for resistance: (**b**,**c**) A, (**d**) B. Numbers in the legend correspond to the value of the frequency of the exciting current.

The frequency behavior of magnetoimpedance was analyzed as a frequency dependence of the maximum value of the MI ratio (ΔR/R_max_) taken from ΔR/R(H) dependencies for each frequency. For the frequency of the 253 MHz value, the maximum of the magnetoimpedance effect was observed (Figure 7a). MI can be used as a method for the definition of an effective magnetic anisotropy field and an effective magnetic anisotropy direction. 

The magnetic anisotropy field corresponded to the maximum MI ratio in the field dependence of the MI ratio for the real part of the impedance. The “two peaks” shape of the MI ratio field dependence corresponded with the magnetic anisotropy along the short side of the MI element [24]. At the frequency of 253 MHz, the magnetic anisotropy field *H_a_* was close to 5 Oe and was directed along the short side of the elements (Figure 7). For the values of skin penetration depth in the range from 600 nm to 300 nm, the corresponding frequency range was from 85 MHz to 400 MHz (Figure 5a), where the magnetic anisotropy field was changed in the range from 5 Oe to 7 Oe (Figure 7c,d). This effect was connected with the inhomogeneity of the magnetic anisotropy field and the dispersion of the EMA over the volume of the elements. As the frequency increased, the skin penetration depth decreased, and the MI signal came from a smaller area near the surface. For example, in paper [31], theoretical calculations without the contribution of the inhomogeneity of the magnetic anisotropy gave non-shifted MI ratio curves.

This possibly pointed out that the surface of elements had a higher magnetic anisotropy field and dispersion of the EMA axis, which was also in accordance with the VSM measurements of *H_a_* = 5 Oe for the whole sample (signal from about one µm thickness) corresponding to the MI measurements for a skin-penetration depth of 600 nm (85 MHz). From the skin-penetration depth of 300 nm (400 MHz), the MI measurements were *H_a_* = 7 Oe corresponding to Kerr microscopy with *H_a_* = 7 Oe, with a signal from thickness at about 20 nm and FMR with Ha: 7 Oe (A) and 10 Oe (B) for signal corresponding to a skin-penetration depth of 180 nm. 

The method of optical-lift off lithography allowed to obtain big batches of MI elements with good repeatable properties, which is important for industrial mass production [27]. Fabricated MI elements showed a maximum MI ratio close to 130% at a frequency of 253 MHz. The maximum sensitivity of 40%/Oe at a frequency of 253 MHz in the range of the external fields from 3 Oe to 5 Oe is quite satisfactory for different applications [45,46,47]. In addition, it is worth mentioning that the MI properties of the elements of the batch (Figure 1a) were rather close to each other with an accuracy of at least 10%. This is a reasonable result, indicating the possibility of the application of a developed fabrication process for the mass production of MI elements under consideration. 

As it mentioned above, in some cases, the operational properties for the real component of the impedance are preferable. In our previous studies [28], the magnetoimpedance effect was analyzed for MI elements of similar types but with a focus on total impedance variation ΔZ/Z. The maximum MI values (ΔZ/Z_max_) of the magnetoimpedance ratio was close to 105% for 169 MHz frequency, i.e., the maximum for total impedance variation was lower in comparison with the real component changes. The maximum sensitivity of 30%/Oe for ΔZ/Z was also lower in comparison with ΔR/R values of about 40%/Oe. Thus, real component detection indeed may have an advantage in certain conditions.

Although we mainly considered the MI effect as a candidate for the possible sensor application of a designed sensitive element, these data can be useful for different electronic components where high magnetic permeability is desired. Properties of the FeNi rectangular elements with different width-to-length ratio and permalloy components with other geometries are currently under special interest in view of their applications as electronic components in many modern devices designed for technological applications [48,49,50,51]. Even more, to some extent, the obtained result can be used for the analysis of magnetostatic interactions in more complex MI composites when size and shape effects become very important or we are dealing with arrays of the elements [51,52,53,54]. 

## 4. Conclusions

Rectangular multilayered magnetoimpedance elements [Cu(3 nm)/FeNi(100 nm)]_5_/Cu(150 nm)/FeNi(10 nm)/Cu(150 nm)/FeNi(10 nm)/Cu(150 nm)/[Cu(3 nm)/FeNi(100 nm)]_5_ were obtained by rf-sputtering and standard lift-off lithographic processes as batches of 12 long (0.5 × 10.0 (mm^2^)) elements. In MI elements, there is competition between transverse magnetic anisotropy, which consist of induced magnetic anisotropy, and magnetostatic interaction between magnetic layers and longitudinal magnetic anisotropy due to shape magnetic anisotropy. 

Magnetostatic properties of MI elements including analysis of the magnetic domain structure indicate the crucial importance of magnetostatic interactions in the analyzed [Cu(3 nm)/FeNi(100 nm)]_5_ multilayered structures. Local parameters of ferromagnetic resonance were measured along the stripe lengths, showing that the deviations of the effective magnetization and the anisotropy field values near the strip edges are related to the orientational inhomogeneity of the magnetization of the MI strip element (being approximately 1 mm from each of the strip edges). The center of the strip can be considered uniform in the angular range ϕ > 10° ÷ 90°, but at ϕ = 0°÷10°, the angular dependence of the resonant field exhibits a deviation from the Stoner–Wohlfarth model. In the region between the strip edges (central 8 mm long part), remarkable homogeneity of the absolute values of *M_eff_* and *H_a_* along the strip length is exhibited, i.e., the detection of an external magnetic field by an element of a given geometry, using magnetoimpedance, is preferable along the long axis of the element, and the length of the element should significantly exceed 2 mm, corresponding with the sum of the length of the edges of the inhomogeneities. 

An obtained linear MI response with respect to the external magnetic field is not near the zero magnetic field, but has a bias effect. Previously, there were attempts to add an antiferromagnetic material to the FeNi multilayer in order to shift the linear magnetic field range to a near zero magnetic field [55]. However, technologically, the usage of a double element configuration might be easier. In any case, the direction to use biasing in MI planar systems is an interesting task to develop in the future.

The MI effect allows to investigate inhomogeneities of magnetic properties within multilayered structures at different thicknesses by varying skin penetration depth with current frequency. The skin penetration depth at FMR is fixed by resonance condition, but this method provides a remarkable quantitative estimation of magnetic inhomogeneities. The maximum sensitivity with respect to the external magnetic field being 40%/Oe at a frequency of 253 MHz in the range of the external fields of 3 Oe to 5 Oe is quite satisfactory for different applications, keeping in mind the advantages of active component R detection in many electronic devices.

## Data Availability

Data available from the corresponding author upon reasonable request.
